# Symmetry structures in dynamic models of biochemical systems

**DOI:** 10.1098/rsif.2020.0204

**Published:** 2020-07-22

**Authors:** Fredrik Ohlsson, Johannes Borgqvist, Marija Cvijovic

**Affiliations:** Department of Mathematical Sciences, Chalmers University of Technology and the University of Gothenburg, 412 96 Gothenburg, Sweden

**Keywords:** ordinary differential equation, model selection, model structure, symmetries

## Abstract

Understanding the complex interactions of biochemical processes underlying human disease represents the holy grail of systems biology. When processes are modelled in ordinary differential equation (ODE) fashion, the most common tool for their analysis is linear stability analysis where the long-term behaviour of the model is determined by linearizing the system around its steady states. However, this asymptotic behaviour is often insufficient for completely determining the structure of the underlying system. A complementary technique for analysing a system of ODEs is to consider the set of symmetries of its solutions. Symmetries provide a powerful concept for the development of mechanistic models by describing structures corresponding to the underlying dynamics of biological systems. To demonstrate their capability, we consider symmetries of the nonlinear Hill model describing enzymatic reaction kinetics and derive a class of symmetry transformations for each order of the model. We consider a minimal example consisting of the application of symmetry-based methods to a model selection problem, where we are able to demonstrate superior performance compared to ordinary residual-based model selection. Moreover, we demonstrate that symmetries reveal the intrinsic properties of a system of interest based on a single time series. Finally, we show and propose that symmetry-based methodology should be considered as the first step in a systematic model building and in the case when multiple time series are available it should complement the commonly used statistical methodologies.

## 1. Introduction

The development of mathematical models is crucial in data-driven fields where the mechanism of the underlying system is of interest. In systems biology, mechanistic models of ordinary differential equations (ODEs) are often constructed to describe the change in abundance of intracellular components such as mRNA or proteins over time. A proposed biological mechanism is typically combined with the law of mass action [1], yielding polynomial models. Under certain assumptions, e.g. regarding the relative abundance of different components, the models can be simplified giving rise to other types of nonlinear rate equations which are common in enzyme kinetics [2]. A classical example is Michaelis–Menten kinetics or, more generally, the Hill equation describing the dynamics of a reaction forming a product, catalysed by an enzyme, in a situation where the concentration of the substrate is substantially higher than that of the enzyme [1]. These rate equations are the building blocks in the construction of mechanistic models in systems biology where each model implicitly proposes an underlying mechanism for the system at hand.

The prevailing strategies for constructing mechanistic models are based on data using a *top down* approach. Given an experimental time series describing the change in the quantity of an intracellular component over time, numerous methods for model selection are based on *residual analysis* [3] using the *least squares* [1] cost function measuring the Euclidean distance between the measured data and the model predictions. Several proposed models are then evaluated and the one that minimizes the cost function is selected. Other more sophisticated methods include the Akaike information criteria [3–5], Bayesian model selection [3–5], cross validation [6,7] and bootstrapping methods [3,5,8] which for example take the model complexity in terms of the number of parameters into account. All these *statistical* methods rely on data (implying that experimental design is an integral part of model selection [9,10]) which limits their applicability in cases when data are scarce or when several models describe the data equally well in terms of the residual analysis, e.g. due to experimental errors which are large compared to the intrinsic variation across candidate models.

Model development can alternatively be conducted using mathematical analysis without any experimental data in a *bottom up* approach. This is traditionally used e.g. in population dynamics [11], where the methodology consists in comparing different mathematical models of the same system [12] in terms of their agreement with properties derived from prior knowledge of the system rather than statistical measures. The most common tool for analysing ODE models in biology is *linear stability analysis* [1,13] where the long-term behaviour of the model is determined by linearizing the system around its steady states. However, this asymptotic behaviour is often insufficient for completely determining the structure of the underlying system. An alternative technique for analysing a system of ODEs is to consider the set of symmetries of its solutions [14–16]. The mathematical framework for such methods is that of group theory and representation theory, and more generally differential geometry. Symmetry methods have been used to classify ODE models according to their symmetry groups [17] and, conversely, identifying symmetries realized in a system allows for a constructive approach to modelling where the symmetries are made manifest in constructing the mechanistic model.

The symmetry framework is well established and enormously successful for model construction in mathematical physics (e.g. as the foundational principle of the standard model of elementary particle physics [18,19]). In fact, it has also found applications within mathematical biology such as animal locomotion [20], and can be used to find solutions to ODEs describing biological systems based on the extensive analysis of nonlinear reaction–diffusion type systems and their symmetries in mathematical physics [21,22]. However, as an approach to construct models in systems biology in general, and reaction kinetics in particular, the symmetry framework is not widely used. Since the framework incorporates intrinsic properties of a system at all time scales, a symmetry-based methodology could arguably represent an untapped potential for systems biology in the context of model development in particular and the analysis of dynamical models in general.

The aim of this paper is to elucidate the role of symmetries in systems biology by demonstrating a minimal example of the application of symmetry methods to model selection in enzyme kinetics. Specifically, provided a time series of the concentration of a substrate of an enzyme over time, and a number of candidate kinetic models describing the data approximately equally well in terms of the residuals, we apply a symmetry-based method to select the model that is best able to represent the time-series data. As the methodology is not commonly used in systems biology, we begin by establishing the framework and deriving a certain class of symmetries of the Hill models used in enzyme kinetics. Subsequently, we evaluate the proposed method applied to model selection among a set of three candidate Hill models. Finally, we discuss the benefits, validity and limitations of the proposed methodology.

## 2. Method

We start by providing a brief theoretical background and introduce the notation that should be sufficient for understanding the implemented symmetry framework. A detailed derivation of the implemented symmetries as well as the methodology for model selection and validation can be found in the electronic supplementary material.

### 2.1. The Hill model

The Hill class of models, describing the enzymatically catalysed conversion of a substrate to a product, are defined by the ODE

2.1 $$\frac{\text{d}S}{\text{d}t}=\Omega_{n}(S,t)$$

with

2.2 $$\Omega_{n}(S,t)=-v_{\max}\frac{S^{n}}{K_{m}+S^{n}},$$

where $n\in N_{+}$ is the order of the model, *S* is the substrate concentration and *t* is the time. The parameters *v*_max_ and *K*_m_, respectively, correspond to the maximum reaction rate and the substrate concentration at half of the maximum reaction rate. In all cases, physical solutions to (equation (2.1)) satisfy *S* ≥ 0 ensuring that *Ω*_*n*_(*S*, *t*) is well defined.

Symmetry properties of the models are most easily analysed in terms of dimensionless time and concentration

2.3 $$\tau =\frac{v_{\max}t}{K_{m}^{1/n}}\;\text{and}\;y=\frac{S}{K_{m}^{1/n}},$$

in terms of which the model (equation (2.1)) becomes

2.4 $$\frac{\text{d}y}{\text{d}\tau }=\omega_{n}(\tau ,y),$$

with

2.5 $$\omega_{n}(\tau ,y)=-\frac{y^{n}}{1+y^{n}}.$$

### 2.2. Symmetry transformations

A point transformation with parameter ϵ∈R acting on the (*τ*, *y*)-plane

2.6 $$\Gamma_{\epsilon }:\;(\tau ,y)\mapsto (\hat{\tau },\hat{y})$$

is a symmetry of the Hill model if it maps a solution of (equation (2.4)) to another solution. In other words, the set of solutions to (equation (2.4)) is closed under the action of a symmetry *Γ*_*ε*_. The family of such symmetry transformations parametrized by *ε* forms a (representation of a) one-parameter Lie group.

There is no time dependence in the expression (equation (2.5)) for the derivative of the concentration, implying that time translation is a manifest symmetry of the theory. The corresponding point transformations are

2.7 $$\Gamma_{\epsilon }:\;(\tau ,y)\mapsto (\tau +\epsilon ,y),$$

under which *ω*_*n*_ are invariant for all model orders *n*.

In the context of elucidating structural properties of a model *ω*_*n*_ from its symmetries, we will also consider point transformations *Γ*_*ε*_ which form a representation of a one-parameter Lie group but which are not symmetries of the model in the sense that the set of solutions is not closed under *Γ*_*ε*_.

It can be shown that the point transformation

**Figure 1.**
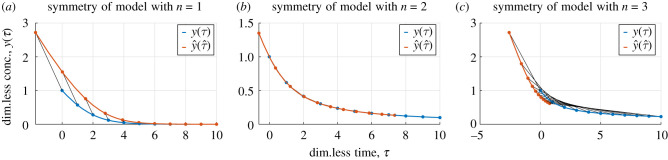
Action of symmetries. The action of the transformation $\Gamma_{\epsilon }^{n}$ in (equation (2.8)) on solutions to the model *ω*_*n*_(*τ*, *y*) for (*a*) *n* = 1, (*b*) *n* = 2 and (*c*) *n* = 3. The action maps a solution *y*(*τ*) (blue) to a different solution $\hat{y}(\hat{\tau })$ (red) for *n* = 1 and *n* = 3. For *n* = 2, the solution is invariant under the action of $\Gamma_{\epsilon }^{2}$ corresponding to symmetry which acts trivially on the space of solutions.

2.8 $$\Gamma_{\epsilon }^{n}:(\tau ,y)\mapsto (-y{\text{e}}^{\epsilon }+(\tau +y){\text{e}}^{-(n-1)\epsilon },y{\text{e}}^{\epsilon })$$

is a symmetry of the Hill model *ω*_*m*_(*τ*, *y*) of order *m* for *m* = *n* but not for *m* ≠ *n*. The symmetry transformation (equation (2.8)) is, therefore, unique to the Hill model of order *n*, which means that it can be used to distinguish between different Hill models. The action of the symmetry transformation on solutions to the Hill models of order *n* = 1, 2, 3 is illustrated in figure 1.

### 2.3. Symmetry-based model selection

Given an experimentally acquired time series and a set of candidate models, the model selection problem consists in determining which candidate model is best able to describe the experimental data, or to conclude that neither of the candidates evaluated represent the data sufficiently well. In situations where several models fit the data approximately equally well in the least-squares sense, they may still be differentiated by the extent to which they capture the global structure of the time series.

One way to achieve a comparison of this structural agreement is by using the fact that the space of solutions to a model is closed under the action of a symmetry transformation of that model, but not under general transformations in (*τ*, *y*)-space. Consequently, the true model generating the time series should have the property that the least-squares error is (approximately) invariant if the following steps are implemented. Initially, a symmetry transformation Γ_*ε*_ is applied to the data, then a model is fitted to the transformed data, the inverse transform is applied to the model and finally the least-squares residuals are computed for the original time series. The invariance is exact in the limit of vanishing errors.

**Figure 2.**
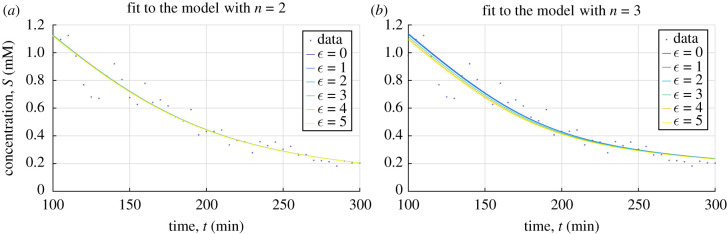
Quality-of-fit to transformed data. Hill models of order *n* = 2, 3 are fitted to data, simulated using a second-order model, after application of the transformation $\Gamma_{\epsilon }^{\;n}$. The inverse transform of the resulting fit (solid lines) is shown for increasing values of the transformation parameter *ε* for (*a*) *n* = 2 and (*b*) *n* = 3. The deterioration of the quality-of-fit for the model *n* = 3 results from $\Gamma_{\epsilon }^{\;3}$ not being a symmetry of the underlying model generating the data.

Conversely, if a symmetry transformation *Γ*_*ε*_ of an incorrect candidate model is applied in the same way the transformation will distort the time series and the quality-of-fit is expected to decrease. In particular, for a one-parameter group of symmetries, we expect that the residuals will increase as a function of the parameter *ε* (at least locally in a neighbourhood of *ε* = 0). The effect on the quality-of-fit resulting from the procedure described above is illustrated in figure 2.

The information about the dependence of the quality-of-fit on the transformation parameter *ε* can be used to complement the information obtained from the ordinary model fitting procedure. Thus, the purpose of the method for model selection described here is not to replace the common approach in systems biology, but rather to augment it using structural information about the candidate models (in the form of their symmetries) to provide additional information regarding their ability to represent a dataset.

### 2.4. Evaluation set-up

To evaluate the symmetry-based model selection methodology, we consider a set-up where a time series of substrate concentrations is simulated using a Hill model of order *n*_Sim_. Subsequently, a number of candidate Hill models, of different orders *n*_Fit_ are fitted to the simulated data using the ‘classical’ least-squares approach and the symmetry-based methodology described above.

The classical approach is based on the *root mean squared (RMS)*, *ρ*_0_ (electronic supplementary material, Eqn. S33 in S5), where the selection criteria is that the model with the best fit, i.e. smallest value of *ρ*_0_, is selected. To calculate the statistical significance of the fitting of the candidate models to a single generated time series, the evaluation procedure is repeated *N* times and confidence intervals of the fits at the one standard error level are calculated. In this setting, models can be distinguished when their confidence intervals are not overlapping.

The symmetry-based methodology is based on the RMS *ρ*(*ε*) (electronic supplementary material, Eqn. S32 in S5) as a function of the transformation parameter *ε*. As in the classic case, confidence intervals of the RMS values are calculated as the evaluation is repeated *N* times. The selection criteria for the symmetry-based methodology is that the model with the lowest RMS-value as *ε* increases is selected where it is required that the confidence intervals of the candidates do not overlap.

It should be noted that it is not obvious what range of the transformation parameter *ε* is required in order to differentiate between the candidate models using the symmetry-based approach, or indeed if it is at all possible. In the examples considered in this paper, the range of *ε* is extended until the RMS curve *ρ*(*ε*) of each model reaches a steady state. If no such state is obtained, the range is extended until convergence becomes prohibitively slow for the nonlinear optimizer or separation of candidate models is considered apparent.

### 2.5. Validation set-up

In order to establish the validity of the symmetry-based methodology, we investigate the case of a point transformation which is not a symmetry for one and only one of the candidate models. The time translation transformation *Γ*_*ε*_ in (equation (2.7)) is a symmetry of all Hill models, and therefore it is expected that the goodness-of-fit is approximately independent of the parameter *ε* for all model orders. We define the *relative RMS*, Δ(*ε*) as

2.9 $$\Delta (\epsilon )=\frac{\rho (\epsilon )}{\rho_{0}}-1,$$

where the value Δ(*ε*) = 0 corresponds to the transformation having no effect on the fitting procedure. Using this metric, the proposed symmetry-based model selection procedure is implemented with the common translation symmetry with the expectation that it will not be possible to distinguish between the various candidates.

## 3. Results

### 3.1. The symmetry-based methodology outperforms residual-based fitting

**Figure 3.**
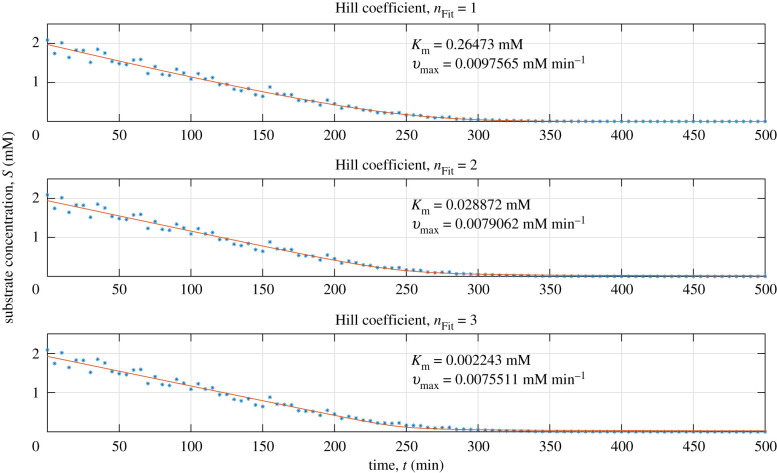
Individual fits of three Hill models. Three candidate models *n*_Fit_ = 1, 2, 3 (red) are fitted to the same simulated time-series data with *n*_Sim_ = 1 (dashed blue) generated using a log-normal error-model with parameters *σ* = 0.1, *v*_max_ = 0.0102 mM min^−1^, *K*_m_ = 0.30 mM and *S*_0_ = 2 mM.

The evaluation procedure described in the previous section is implemented for three cases, namely *n*_Sim_ = 1, 2, 3. For each case, the three candidate models with *n*_Fit_ = 1, 2, 3 are fitted to a simulated time series using both the classical and the symmetry-based method. The procedure is repeated *N* = 5 times and the corresponding confidence intervals are calculated. For the implemented noise levels and values of the kinetic parameters, i.e. *v*_max_ and *K*_m_, used in the simulations, the classical quality-of-fit is similar for all candidate models (figure 3).

**Figure 4.**
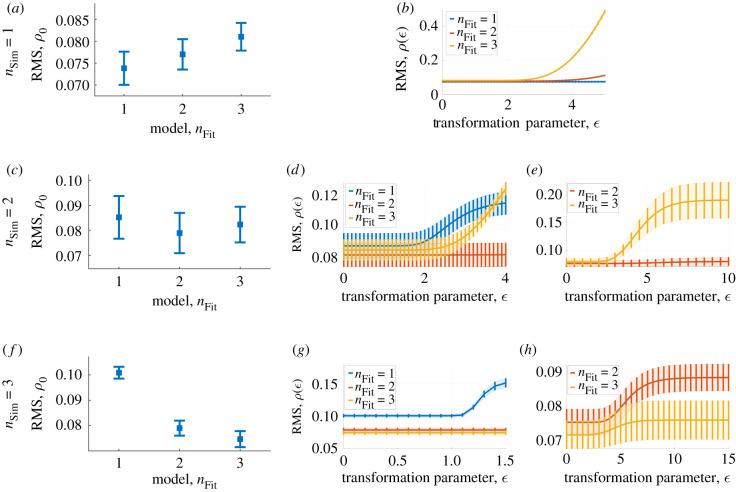
Model selection with distinct symmetries compared to the classic residual-based approach. From the top to the bottom row, the data are generated with *n*_Sim_ = 1, 2, 3 using a log-normal error-model with parameters *σ* = 0.1, *v*_max_ = 0.0102 mM min^−1^, *K*_m_ = 0.30 mM and *S*_0_ = 2 mM. (*a*) The residual-based RMS measure *ρ*_0_ fails to significantly distinguish between the *n*_Fit_ = 1, 2 models, but rejects the *n*_Fit_ = 3 model, for the datasets with *n*_Sim_ = 1. (*b*) Over the range *ε* ∈ [0, 5] the symmetry-based RMS measure *ρ*(*ε*) indicates that *n*_Fit_ = 1 is significantly better than *n*_Fit_ = 2, 3 for the datasets with *n*_Sim_ = 1. (*c*) The residual-based RMS measure *ρ*_0_ fails to significantly distinguish between the *n*_Fit_ = 1, 2, 3 models for the datasets with *n*_Sim_ = 2. (*d*) Over the range *ε* ∈ [0, 4], the symmetry-based RMS measure *ρ*(*ε*) indicates that *n*_Fit_ = 2 is significantly better than *n*_Fit_ = 1, 3 for the datasets with *n*_Sim_ = 2. (*e*) Over the range *ε* ∈ [0, 10], the symmetry-based RMS measure *ρ*(*ε*) rejects the model with *n*_Fit_ = 3 and selects the model with *n*_Fit_ = 2 for the datasets with *n*_Sim_ = 2. (*f*) The residual-based RMS measure *ρ*_0_ fails to significantly distinguish between the *n*_Fit_ = 2, 3 models but it can reject the first model with *n*_Fit_ = 1 for the datasets with *n*_Sim_ = 3. (*g*) Over the range *ε* ∈ [0, 1.5], the symmetry-based RMS measure *ρ*(*ε*) draws the same conclusion as the classic approach based on *ρ*_0_ in (*f*). In other words, the model with *n*_Fit_ = 1 is rejected while the methodology cannot distinguish between the *n*_Fit_ = 2, 3 models for the datasets with *n*_Sim_ = 3. (*h*) Over the range *ε* ∈ [0, 15], the symmetry-based RMS measure *ρ*(*ε*) rejects the model with *n*_Fit_ = 2 and selects the model with *n*_Fit_ = 3 for the datasets with *n*_Sim_ = 3.

For the Hill model of order *n* = 1 (i.e. *n*_Sim_ = 1), the classical approach cannot distinguish between the fitted models of order *n* = 1, 2 (i.e. *n*_Fit_ = 1, 2) as the confidence intervals of the RMS *ρ*_0_ overlap (figure 4*a*). However, the symmetry-based methodology clearly rejects the models with *n*_Fit_ = 2, 3 and selects the true model with *n*_Fit_ = 1 on the interval *ε* ∈ [0, 5] (figure 4*b*).

In the case of the Hill model of order *n* = 2 (i.e. *n*_Sim_ = 2), the classical approach cannot distinguish between the models, as the confidence intervals of the RMS fitting overlap (figure 4*c*), while the symmetry-based methodology again selects the true model. Over the range *ε* ∈ [0, 4] the confidence intervals of the various models clearly separate using the symmetry-based methodology (figure 4*d*) and the correct model with *n*_Fit_ = 2 is selected as it has the lowest RMS-value *ρ*(*ε*). In fact, this effect is exaggerated when the range of the transformation parameter is increased to *ε* ∈ [0, 10] (figure 4*e*) and it is evident in this case that the true model would be selected using the symmetry-based approach.

As in the previous cases, the symmetry-based methodology outperforms the classical approach even for the Hill model of order *n* = 3 (i.e. *n*_Sim_ = 3). The classical approach rejects the first model with *n*_Fit_ = 1 while it cannot distinguish between the *n*_Fit_ = 2, 3 models as their confidence intervals overlap (figure 4*f*). For a short range of the transformation parameter *ε* ∈ [0, 1.5], the symmetry-based methodology reaches the same conclusion (figure 4*g*). Thus, for small values of the transformation parameter *ε* the symmetry-based methodology rejects the first model while it cannot distinguish between the other models as their confidence intervals of *ρ*(*ε*) overlap. However, by increasing the range of the transformation parameter to *ε* ∈ [0, 15] it is clear that the true model with *n*_Fit_ = 3 is selected and that the incorrect model with *n*_Fit_ = 2 is rejected (figure 4*h*).

### 3.2. The translation symmetry cannot distinguish between models

As expected, the common translation symmetry does not distinguish between the candidate models. For all three datasets generated with the models *n*_Sim_ = 1, 2, 3, the relative RMS Δ(*ε*) is zero within numerical errors. Accordingly, the manifest translation transformation (equation (2.7)) is incapable of distinguishing between the candidate models *n*_Fit_ = 1, 2, 3. This result validates the fundamental assumption of the symmetry-based methodology, namely that the symmetries Γ_*ε*_ of the candidate models must be distinct in order to differentiate between them. Furthermore, it provides a consistency check of the method by using a symmetry transformation different from the specific transformations $\Gamma_{\epsilon }^{n}$ in (equation (2.8)) used to generate the results in figure 4. The details of the validation of the methodology is provided in electronic supplementary material, S6.

## 4. Discussion

The construction, analysis and validation of mechanistic models of complex cellular processes represent the heart of systems biology. We present a minimal example of the use of symmetries on the very building blocks of kinetic modelling in systems biology, by deriving symmetries of the Hill equation. Moreover, we demonstrate that symmetries reveal intrinsic properties of a system of interest by presenting an example of a methodology for selecting Hill models based on a single time series. In fact, with one time series of substrate concentration over time no single candidate model corresponding to the fitted Hill models (*n*_Fit_ = 1, 2, 3) can be identified using classical model fitting while the symmetry-based methodology identifies the correct one in all cases (figure 4). We also validate the underlying assumption of the methodology, namely that the symmetries of the candidate models must be distinct, by implementing the common translation symmetry in the proposed methodology (see electronic supplementary material, figure S3). Thus, this provides a minimal example of the fact that symmetries can be used to deduce intrinsic properties of a system where few data are available in a way that regular model fitting cannot.

Importantly, the symmetry-based model selection is not based on the assumption that any of the candidate models is in fact the correct model of the underlying system. If all evaluated transformations *Γ*_*ε*_ cause a significant increase in *ρ* with the transformation parameter *ε*, or if no steady state is reached, the conclusion is to reject all of the transformations at hand as symmetries of the system. Similarly, if several models reach a steady state with negligible increase in the error they are the both to be considered viable candidates for describing the underlying process, and the symmetry method cannot distinguish between them. Conversely, if several symmetries are identified any mathematical model of the system must respect all of them. Therefore, the symmetry-based evaluation described in the present paper should more accurately be considered as the first step in a systematic model construction as opposed to simply a methodology for selecting among candidate models.

In cases where multiple time series are available, it is possible to estimate the log-likelihood function and thereby use statistical methods such as the AIC or BIC criteria for model selection. However, if the actual underlying mechanisms of the studied system is of interest then symmetries can still provide novel insights that classical model selection methodologies cannot. Accordingly, symmetries are not meant to replace the already existing statistical methodologies for model selection but rather to complement them in the construction of mechanistic models.

Although the symmetry principle applied in the example discussed in the present paper extends other classes of models and to arbitrary values of the Hill coefficient *n*, the simple implementation of the principle in our algorithm is not expected to be generally applicable. In particular, the dynamics of the Hill model becomes faster as *n* increases and its solutions correspondingly more nonlinear. Accommodating this fact, or similar effects in dynamical systems beyond the Hill model, might require modifications to the cost function used in the curve fitting optimization problem or even the introduction of a non-trivial transformation of the data series in order to make it amenable to symmetry analysis.

Similarly, the symmetry method is expected to be applicable to more complex models, such as systems of ODEs, but requires an extended analysis and implementation. As for the case of a single ODE, symmetries specific to the system at hand must be explicitly constructed for each case, and the higher level of complexity of the dynamics requires more sophisticated algorithms to be implemented. The extension of the analysis presented in the present paper to more general models of (systems of) ODEs constitutes a potential direction for future research.

A further natural continuation of the work presented here is the automatization of the methodology for identifying model symmetries in an algorithmic fashion. This would entail the usage of numerical combined with algebraic methods [16], in order to allow for systematic model structure identification for larger models describing the dynamics of e.g. large intracellular pathways. Such an automatization requires the formulation of a criteria for the range of the transformation parameter *ε* in order to determine whether or not a certain transformation constitutes a symmetry. In addition, the methodology relies on Taylor expansions locally around *ε* ≈ 0 and it is not evident when the derived transformation ceases to be accurate. Furthermore, as discussed above, the range of the transformation parameter is crucial when using the symmetry-based methodology as a means of selecting one model among multiple candidates. For example, over the range *ε* ∈ [0, 1.5] in the case of the dataset generated with the model with *n*_Sim_ = 3, the methodology cannot distinguish between the models with *n*_Fit_ = 2 and *n*_Fit_ = 3 (figure 4*g*) while over the range *ε* ∈ [0, 15] (figure 4*h*) the correct model corresponding to *n*_Fit_ = 3 is selected and the incorrect model with *n*_Fit_ = 2 is rejected.

As the ultimate goal of systems biology is to gain comprehensive mechanistic understanding of how complex functions emerge from the interaction of biomolecules, symmetries constitute a forceful constituent in modelling where the underlying process is of interest. As fundamental properties of a given system can be described by their corresponding symmetries, where energy conservation corresponds to invariance under time translations, it is of interest to be able to deduce the symmetries governing the system directly from the available data. By studying which symmetries a system obeys, it is possible to derive the corresponding dynamic models from those symmetries [15,16,23,24]. This study serves as an example of how this very potent methodology can be introduced into dynamic modelling in systems biology. As the symmetry framework is well established in physics, the prospects of constructing, understanding and analysing models using symmetries in systems biology are exciting.

## Supplementary Material

Detailed derivation of the presented method

## Data accessibility

This article has no additional data.
