# Supplementary material for: Symmetry structures in dynamic models of biochemical systems
Source: J R Soc Interface. 2020 Jul 22;17(168):20200204. doi: 10.1098/rsif.2020.0204 (PMC7423443; doi:10.1098/rsif.2020.0204)
Supplement: Detailed derivation of the presented method [file rsif20200204supp1.pdf]

Supplementary material to the article

Symmetry structures in dynamic models of biochemical systems

Fredrik Ohlsson*, Johannes Borgqvist*, Marija Cvijovic

Department of Mathematical Sciences
Chalmers University of Technology and the University of Gothenburg
SE-412 96 Gothenburg, Sweden

Contents

S1 The Hill model **S1**

S2 Simulation methodology for generating the data **S2**

S3 Symmetries of first order ODE's **S4**

S4 Symmetries of the Hill model **S5**

S5 Model selection method **S7**

S6 Validation using the translation symmetry **S10**

List of Figures

S1 Simulated time series S3
S2 Illustration of the proposed method S9
S3 Method validation using the translation symmetry S10

fredrik.ohlsson@chalmers.se
johborgq@chalmers.se
marija.cvijovic@chalmers.se
*Both authors contribute equally.

S1 The Hill model

The Hill model describes the conversion of a substrate S into a product P. The reaction is catalysed by an enzyme E where the substrate binds to an active site of the enzyme forming a substrate-enzyme complex C. The complex then forms the product from the substrate in an irreversible reaction before dissociating from the product. In the general setting, it is possible to assume that an enzyme has $n \in \mathbb{N}_+$ active sites corresponding to the assumption that n substrate units are required in order to form one unit of the product. Furthermore, under certain conditions it is possible to assume that it is the binding of the first substrate unit that is the *rate-limiting step*, implying that as soon as the first substrate unit binds to the enzyme the other units bind immediately. These conditions are described from a kinetic point of view by the reactions

$$n\,S + E \underset{k_{-1}}{\overset{k_1}{\rightleftharpoons}} C \xrightarrow{k_2} P + E\,.$$

In the above reactions, k_1 is the rate constant for the binding of the substrate units to the enzyme, k_{-1} is the rate constant for the dissociation of the substrate units from the enzyme and k_2 is the rate constant describing the conversion of the substrate units to the product. Assuming that the *law of mass action* holds, the dynamics of these reactions is governed by

$$\begin{cases} \frac{\mathrm{d}S}{\mathrm{d}t} &= -k_1 S^n E &+ & k_{-1}C \\ \frac{\mathrm{d}E}{\mathrm{d}t} &= -k_1 S^n E &+ & (k_{-1}+k_2)C \\ \frac{\mathrm{d}C}{\mathrm{d}t} &= k_1 S^n E &- & (k_{-1}+k_2)C \\ \frac{\mathrm{d}P}{\mathrm{d}t} &= k_2 C & & \end{cases} \tag{S1}$$

with initial conditions

$$S(0) = S_0 \quad , \quad E(0) = E_{\mathrm{tot}} \quad , \quad C(0) = 0 \quad , \quad P(0) = 0\,. \tag{S2}$$

Note that *the total number of enzymes* $E_{\mathrm{tot}} = E + C$ appears as the initial condition for the unbound form of the enzyme E since there is no complex initially and it is clear that E_{tot} is conserved

$$\frac{\mathrm{d}E_{\mathrm{tot}}}{\mathrm{d}t} = \frac{\mathrm{d}E}{\mathrm{d}t} + \frac{\mathrm{d}C}{\mathrm{d}t} = 0\,. \tag{S3}$$

Assuming that the amount of enzyme is much smaller than the amount of substrate, i.e. $S_0 \gg E_{\text{tot}}$, implying that the enzymes are always saturated, it is possible to motivate the assumption

$$\frac{\mathrm{d}E}{\mathrm{d}t} = \frac{\mathrm{d}C}{\mathrm{d}t} = 0\,. \tag{S4}$$

Substituting this equation into the system of equations above yields

$$\frac{\mathrm{d}S}{\mathrm{d}t} = -v_{\max}\left(\frac{S^n}{K_m + S^n}\right) \quad , \quad S(0) = S_0\,, \tag{S5}$$

which is referred to as the *Hill equation*, in which we have introduced the constants

$$K_{\mathrm{m}} = \frac{k_{-1} + k_2}{k_1} \quad , \quad v_{\max} = k_2 E_{\text{tot}} \tag{S6}$$

and the number of active sites $n \in \mathbb{N}_+$ is called the *Hill coefficient*, or the order of the Hill model.

The non-dimensionalisation of the Hill equation is obtained by introducing

$$\tau = \frac{v_{\max} t}{K_{\mathrm{m}}^{1/n}} \quad , \quad y = \frac{S}{K_{\mathrm{m}}^{1/n}} \tag{S7}$$

and substituting these two dimensionless components into the original model (Eq S5) yields the following dimensionless version of the equation describing the consumption of the substrate

$$\frac{\mathrm{d}y}{\mathrm{d}\tau} = -\frac{y^n}{1 + y^n} \quad , \quad y(0) = y_0 = \frac{S_0}{K_{\mathrm{m}}^{1/n}}\,. \tag{S8}$$

It is worth emphasising that the only parameter in the dimensionless model (Eq S8) is the initial condition y_0.

S2 Simulation methodology for generating the data

In simulating data for the Hill model, we have used kinetic parameters for the enzyme β-lactamase I from the organism *Bacillus cereus* [S1, S3]. The reported parameters corresponding to the full enzymatic system (Eq S1) are

$k_1 = 0.068$ mM^{-1}min^{-1}, $k_{-1} = 0.0136$ min^{-1} and $k_2 = 0.0068$ min^{-1}. In the simplified Hill model (Eq S5) this corresponds to a value of $K_\mathrm{m} = 0.30$ mM and we have implemented a value of $E_\mathrm{tot} = 1.5$ mM for the total enzyme concentration resulting in the maximal reaction rate $v_\mathrm{max} = 0.0102$ mM min^{-1}. All simulations use an initial substrate concentration of $S_0 = 2$ mM and a log-normal error-model

$$S(t) = S_\mathrm{H}(t)e^{\eta}, \quad \eta \sim \mathcal{N}(0, \sigma)\,. \tag{S9}$$

In the above equation, $S(t)$ corresponds to the simulated data at time t, $S_\mathrm{H}(t)$ corresponds to the underlying process at time t, given by the solution the ODE-model (Eq S5), and η is the error drawn from a normal distribution with standard deviation σ. In all simulations, we have implemented a noise level of 10%, i.e. $\sigma = 0.1$. An example of simulated data is shown in Fig S1.

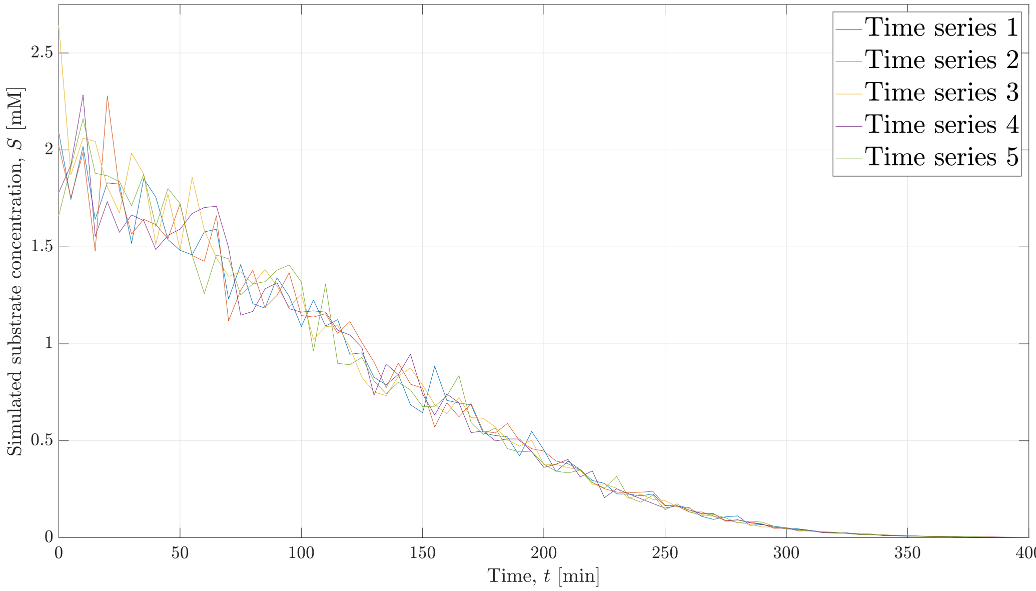

Figure S1. **Simulated time series of substrate concentration**. Five time series with substrate concentration over time are presented. The data is simulated with a log-normal error-model with parameters $\sigma = 0.1$, $v_\mathrm{max} = 0.0102$ mM min^{-1}, $K_\mathrm{m} = 0.30$ mM and $S_0 = 2$ mM.

The choice to implement a log-normal error-model (Eq S9), as opposed to a simpler additive error-model, requires some motivation. Firstly, for con-

centrations close to zero it is possible to obtain unphysical negative values of S with an additive model which is avoided with the log-normal model. Secondly, in applications it is often the case that errors associated with measurements for high concentrations are larger than the corresponding measurement-errors for low concentrations [S2]. This effect is captured in the log-normal error-model but not in the additive model, in which the absolute errors are of equal size across the entirety of the time series.

S3 Symmetries of first order ODE's

In this section, we summarise the general theory for Lie symmetries of a single first order ODE

$$\frac{\mathrm{d}y}{\mathrm{d}\tau} = \omega(\tau, y)\,. \tag{S10}$$

A solution of (Eq S10) is a curve $y(\tau)$ in the (τ, y) plane and a point transformation is a map $\Gamma : \mathbb{R}^2 \to \mathbb{R}^2$ defined by its action on an arbitrary point

$$\Gamma : (\tau, y) \mapsto (\hat{\tau}(\tau, y), \hat{y}(\tau, y))\,. \tag{S11}$$

A point transformation constitutes a symmetry of the ODE (Eq S10) if it maps the set of solutions to itself, that is if

$$\frac{\mathrm{d}\hat{y}}{\mathrm{d}\hat{\tau}} = \omega(\hat{\tau}, \hat{y}) \quad \text{if} \quad \frac{\mathrm{d}y}{\mathrm{d}\tau} = \omega(\tau, y)\,. \tag{S12}$$

We consider exclusively sets of symmetry transformations,

$$\Gamma_\epsilon : (\tau, y) \mapsto (\hat{\tau}, \hat{y})\,, \tag{S13}$$

parameterised by a number $\epsilon \in \mathbb{R}$, which are diffeomorphisms of $\mathbb{R}^2$ and form a representation of a (local) one-parameter Lie group G. For such representations Γ_0 is the trivial transformation, and there exists a neighbourhood U of $\epsilon = 0$ such that $\Gamma_\delta \Gamma_\epsilon = \Gamma_{\delta+\epsilon}$ for $\delta, \epsilon \in U$ and $\hat{\tau}$ and $\hat{y}$ can be represented as Taylor series in ϵ in U. In particular, this implies that the inverse of a transformation is obtained as

$$\Gamma_\epsilon^{-1} = \Gamma_{-\epsilon}\,. \tag{S14}$$

The set of points $(\hat{\tau}, \hat{y})$ obtained by the action of the Lie group G on (τ, y) is called the orbit of (τ, y). At the point $(\hat{\tau}, \hat{y})$ the vector tangent to the orbit

is $(\xi(\hat\tau,\hat y),\eta(\hat\tau,\hat y))$ where

$$\xi(\hat\tau,\hat y) = \frac{\mathrm{d}\hat\tau}{\mathrm{d}\epsilon} \quad , \quad \eta(\hat\tau,\hat y) = \frac{\mathrm{d}\hat y}{\mathrm{d}\epsilon} \,. \tag{S15}$$

In particular, the existence of a Taylor series expansion around $\epsilon = 0$ implies that

$$\hat\tau = \tau + \epsilon\xi(\tau,y) + \mathcal{O}(\epsilon^2) \quad , \quad \hat y = y + \epsilon\eta(\tau,y) + \mathcal{O}(\epsilon^2) \tag{S16}$$

with

$$\xi(\tau,y) = \left.\frac{\mathrm{d}\hat\tau}{\mathrm{d}\epsilon}\right|_{\epsilon=0} \quad , \quad \eta(\tau,y) = \left.\frac{\mathrm{d}\hat y}{\mathrm{d}\epsilon}\right|_{\epsilon=0} \,. \tag{S17}$$

Since the symmetry transformations Γ_ϵ are diffeomorphisms of $\mathbb{R}^2$, the assignment of (ξ,η) is smooth and defines a vector field on $\mathbb{R}^2$ by

$$X = \xi(\tau,y)\partial_\tau + \eta(\tau,y)\partial_y \,. \tag{S18}$$

The vector field generates the symmetry transformation Γ_ϵ through

$$\hat\tau = e^{\epsilon X}\tau \quad , \quad \hat y = e^{\epsilon X} y \,, \tag{S19}$$

where $e^{\epsilon X}$ is the equivariant exponential map satisfying

$$\Phi(e^{\epsilon X}\tau, e^{\epsilon X}y) = e^{\epsilon X}\Phi(\tau,y) \tag{S20}$$

for an arbitrary function $\Phi : \mathbb{R}^2 \to \mathbb{R}$. The vector field X, called the infinitesimal generator of Γ_ϵ, contains all information required to reconstruct the corresponding transformations.

Consequently, the equation (Eq S10) admits a one-parameter Lie group of symmetries generated by (Eq S18) if $\xi(t,y)$ and $\eta(t,y)$ satisfy the linearised symmetry condition

$$\partial_\tau\eta + (\partial_y\eta - \partial_\tau\xi)\omega - \partial_y\xi\omega^2 = \xi\partial_\tau\omega + \eta\partial_y\omega \,. \tag{S21}$$

S4 Symmetries of the Hill model

The class of symmetries primarily considered in the present paper are obtained by using an Ansatz linear in both τ and y for the components $\xi(\tau,y)$ and $\eta(\tau,y)$ of the generating vector field X, according to

$$\xi(\tau,y) = A\tau + By \quad , \quad \eta(\tau,y) = C\tau + Dy \tag{S22}$$

for the Hill model $\omega_n(\tau, y)$ in (Eq S8). The linearised symmetry condition (Eq S21) takes the form

$$ny^{n-1}\left(C\tau + Dy\right) + \left(1+y^n\right)^2 C - y^n\left(1+y^n\right)\left(D-A\right) - y^{2n}B = 0 \quad \text{(S23)}$$

which for $n \in \mathbb{N}_+$ has the general solution

$$A = -(n-1)\lambda \quad , \quad B = -n\lambda \quad , \quad C = 0 \quad , \quad D = \lambda \, , \quad \text{(S24)}$$

where $\lambda \in \mathbb{R}$ is an arbitrary constant. Up to simultaneous rescalings, the components of the tangent vector are therefore

$$\xi(\tau, y) = -(n-1)\tau - ny \quad , \quad \eta(\tau, y) = y \quad \text{(S25)}$$

and the corresponding generating vector field is given by

$$X = -\left((n-1)\tau + ny\right)\partial_\tau + y\partial_y \, . \quad \text{(S26)}$$

The coordinate transformation generated by (Eq S26) is obtained by the exponential map as

$$(\hat{\tau}, \hat{y}) = (-ye^{\epsilon} + (\tau + y)e^{-(n-1)\epsilon}, ye^{\epsilon}) \quad \text{(S27)}$$

where ϵ is the transformation parameter.

In addition to the linear symmetries described above, which are different for models of different values of n, the Hill models (Eq S8) are all manifestly invariant under a time translation transformation

$$(\hat{\tau}, \hat{y}) = (\tau + \epsilon, y) \quad \text{(S28)}$$

for all $n \in \mathbb{N}_+$. The transformation is generated by the vector field

$$X = \partial_\tau \, , \quad \text{(S29)}$$

with components $\xi(\tau, y) = 1$ and $\eta(\tau, y) = 0$, which trivially satisfy the linearised symmetry condition since $\partial_\tau \omega_n = 0$. Since the Hill model is a first order ODE, its solution is uniquely determined by a choice of initial condition at (say) $\tau = 0$. Consequently, any non-trivial symmetry transformation can be obtained as equivalent time translation with a suitable choice of transformation parameter.

S5 Model selection method

The purpose of the symmetry based method for model selection is to incorporate global structural properties of the time series in the comparison of candidate models. Symmetry transformations preserve the space of solutions to the ODE model, whereas general coordinate transformations do not. Consequently, if we apply a symmetry transformation Γ_ϵ of the candidate model $\omega(\tau, y)$ to the (non-dimensionalised) time series data, perform a least square fit to the transformed data to obtain a solution of the candidate model, and then apply the inverse transformation $\Gamma_{-\epsilon}$ to the fitted model, the result will be a (different) solution to the model $\omega(\tau, y)$. If the transformation Γ_ϵ is also a symmetry of the model generating the time series data, the residuals between the original time series data and the resulting solution should be approximately independent of ϵ. In the limit of vanishing errors in the time series data the independence becomes exact.

Conversely, if a transformation Γ_ϵ which is not a symmetry of the true model is applied in the same way, it will distort the transformed time series data, causing a reduction in the quality-of-fit of the candidate model. The dependence of the residuals on the transformation parameter ϵ is non-linear, but in a neighbourhood of $\epsilon = 0$ we expect the residuals of the fit to be increasing since $\epsilon = 0$ corresponds to the trivial transformation which introduces no distortion. Throughout this paper, we use the ordinary RMS error, $\rho(\epsilon)$ (Eq S32), for the residuals to quantify the quality-of-fit.

Since the symmetries of each candidate Hill model are most conveniently implemented in the dimensionless coordinates (τ, y) defined in (Eq S7), a preliminary step in the model selection process outlined above is to estimate the values of K_{m} and $v_{\max}$ from the simulated data $\{(t_i, S_i)\}_{i=1}^{N}$, using ordinary nonlinear least-square optimisation, and compute the non-dimensionalised data $\{(\tau_i, y_i)\}_{i=1}^{N}$.

In order to avoid introducing a dependence on the non-dimensionalisation, which differs depending on the candidate model, $\rho(\epsilon)$ is always computed in the original dimensional context. Furthermore, to obtain a meaningful comparison between the effects of symmetry transformations of different candidate models we also normalise the scale of the parameter ϵ so that $\epsilon = 1$ corresponds to the initial data point (τ_1, y_1) being shifted by (at least) 50% of the time series range in both the τ and the y direction,

$$|\hat{\tau}_1 - \tau_1| \geq \frac{|\tau_N - \tau_1|}{2} \quad , \quad |\hat{y}_1 - y_1| \geq \frac{|y_N - y_1|}{2} \, , \tag{S30}$$

where $(\hat{\tau}_1, \hat{y}_1)$ is the result of applying $\Gamma^n_{\epsilon=1}$ to (τ_1, y_1).

Using the fact that the solution to any first order ODE is uniquely determined by a choice of initial condition, we introduce the notation

$$H_n(\tau \mid \tau_0, y_0) = \left\{ y \in \mathcal{C}^1 \;\middle|\; \frac{\mathrm{d}y}{\mathrm{d}\tau} = \omega_n(\tau, y)\,,\; y(\tau_0) = y_0 \right\} \tag{S31}$$

for a solution to the Hill model of order $n \in \mathbb{N}_+$. Given the time series $\{(t_i, S_i)\}_{i=1}^N$ of substrate concentrations, a set of integers n defining the candidate Hill models and a corresponding set of representations Γ^n_ϵ of one-parameter symmetry groups unique to each candidate model, the method for symmetry based Hill model selection can be described as follows. For each value n of the candidate model order:

1. Estimate parameters K_{m} and $v_{\max}$ from $\{(t_i, S_i)\}_{i=1}^N$
2. Compute non-dimensionalised time series $\{(\tau_i, y_i)\}_{i=1}^N$
3. Normalise transformation parameter ϵ
4. For each value ϵ of the transformation parameter:

   i. Apply transformation Γ^n_ϵ to the data
   $$\{(\hat{\tau}_i, \hat{y}_i)\}_{i=1}^N = \{\Gamma^n_\epsilon(\tau_i, y_i)\}_{i=1}^N$$

   ii. Set $\hat{\tau}_0 = \hat{\tau}_1$ and determine least-square fit $\hat{H}_n(\tau \mid \hat{\tau}_0, \hat{y}_0)$ from
   $$\hat{y}_0 = \arg\min_{\xi} \sum_{i=1}^{N} \left(H_n(\hat{\tau}_i \mid \hat{\tau}_0, \xi) - \hat{y}_i\right)^2$$

   iii. Apply the inverse transform $\Gamma^n_{-\epsilon}$ to the model
   $$H_n(\tau \mid \tau_0, y_0) = \hat{H}_n\left(\tau \mid \Gamma^n_{-\epsilon}(\hat{\tau}_0, \hat{y}_0)\right)$$

   iv. Evaluate the fit $\rho(\epsilon)$ of H_n to $\{(\tau_i, y_i)\}_{i=1}^N$
   $$\rho(\epsilon) = K_{\mathrm{m}}^{1/n} \left(\sum_{i=1}^{N} \frac{1}{N} \left(H_n(\tau_i \mid \tau_0, y_0) - y_i\right)^2 \right)^{1/2} \tag{S32}$$

The different parts of Step 4 of the method are illustrated in Fig S2.

Using the function $\rho(\epsilon)$ we can express the ordinary RMS error of the classical approach as

$$\rho_0 = \rho(0)\,. \tag{S33}$$

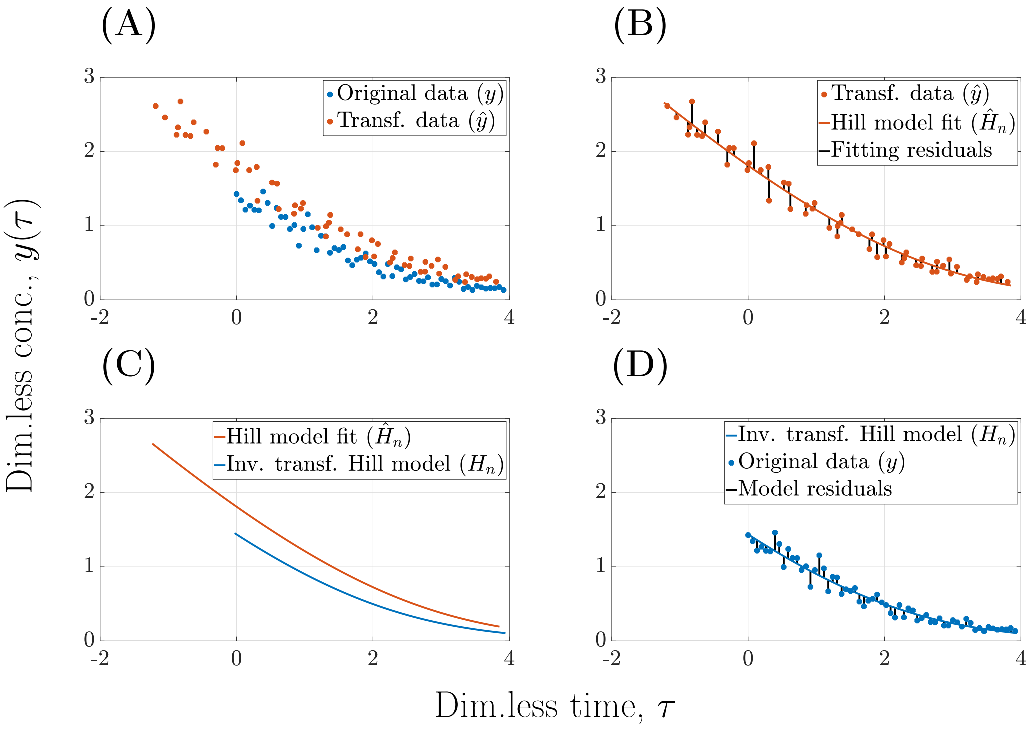

Figure S2. **Illustration of the symmetry based method for model selection**. The simulated dimensionless concentration $y(\tau)$ plotted versus dimensionless time τ for the $n = 1$ model. The point transformation implemented is the symmetry Γ^1_ϵ of the model. **(A)** The original (y) and the transformed ($\hat{y}$) time series. **(B)** The transformed time series ($\hat{y}$), the fitted Hill model ($\hat{H}_n$) and the corresponding residuals. **(C)** The fitted Hill model ($\hat{H}_n$) and its inverse transform (H_n). **(D)** The inverse of the fitted Hill model (H_n), the original data (y) and the corresponding residuals.

S6 Validation using the translation symmetry

As described in the article, the validity of the proposed methodology is tested using the common translation transformation (Eq 7). Recall that the *relative RMS*, $\Delta(\epsilon)$ was defined to this end as follows

$$\Delta(\epsilon) = \frac{\rho(\epsilon)}{\rho_0} - 1\,, \tag{S34}$$

where the value $\Delta(\epsilon) = 0$ corresponds to the transformation having no effect.

For all three data sets generated with the models $n_{\mathrm{Sim}} = 1, 2, 3$ the confidence intervals of the relative RMS is of the order 10^{-12} centered around $\Delta = 0$ (Fig S3). Numerical tolerance of the optimiser is set to 10^{-12} which suggests that $\Delta(\epsilon)$ is zero to within numerical errors. Consequently, the translation symmetry cannot distinguish between the candidate models.

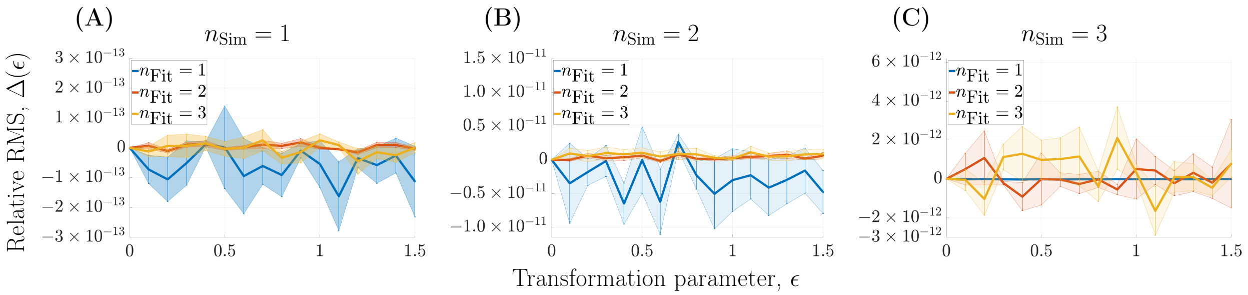

Figure S3. **Validation of the symmetry based approach using the translation symmetry**. In all three cases, the models with $n_{\mathrm{Fit}} = 1, 2, 3$ are fitted to the simulated data over the range $\epsilon \in [0, 1.5]$. The relative RMS $\Delta(\epsilon)$ is plotted against the transformation parameter ϵ where the model selection is conducted with the common translation symmetry $\Gamma_\epsilon : (t, y) \mapsto (\hat{t}, \hat{y}) = (t + \epsilon, y)$. The data is generated with the models corresponding to **(A)** $n_{\mathrm{Sim}} = 1$, **(B)** $n_{\mathrm{Sim}} = 2$ and **(C)** $n_{\mathrm{Sim}} = 3$ respectively. The data is generated using a log-normal error-model with parameters: $\sigma = 0.1$, $v_{\max} = 0.0102$ mM min^{-1}, $K_{\mathrm{m}} = 0.30$ mM and $S_0 = 2$ mM. In all cases, the methodology cannot distinguish between any of the models as the relative RMS is within the range of the numerical tolerance, i.e $|\Delta(\epsilon)| \approx 10^{-12}$. This result confirms the fact that Γ_ϵ is a symmetry of all three models.

References Supplementary

[S1] Roy Bicknell and Stephen G Waley. Single-turnover and steady-state kinetics of hydrolysis of cephalosporins by β-lactamase i from bacillus cereus. *Biochemical Journal*, 231(1):83–88, 1985.

[S2] Stig Bousgaard Mortensen, Anna Helga Jónsdóttir, Søren Klim, and Henrik Madsen. Introduction to pk/pd modelling-with focus on pk and stochastic differential equations. 2008.

[S3] Márcio A Mourão, Jeyaraman Srividhya, Patrick E McSharry, Edmund J Crampin, and Santiago Schnell. A graphical user interface for a method to infer kinetics and network architecture (mikana). *PloS one*, 6(11), 2011.
